# Effectiveness of Structured Exercise Programs in Patients With Conduction System Pacing and Cardiac Resynchronization Therapy: A Systematic Review

**DOI:** 10.7759/cureus.112023

**Published:** 2026-07-03

**Authors:** Kiran S Dhaygude, Poovishnu T Devi, Rushikesh S Patil, Janhavee S Brahmadande

**Affiliations:** 1 Department of Cardiopulmonary Sciences, Krishna College of Physiotherapy, Krishna Vishwa Vidyapeeth (Deemed to be University), Karad, IND

**Keywords:** cardiopulmonary, functional capacity, pacemaker, physiotherapy, rehabilitation

## Abstract

Following the implantation of cardiac physiological pacemakers and similar electronic cardiac devices, individuals frequently face a decline in physical activity levels, marked exercise intolerance, compromised functional capacities, and a diminished overall quality of life. These constraints are often aggravated by an underlying fear of physical exertion, pre-existing myocardial dysfunction, bodily deconditioning, and various psychosocial barriers. Consequently, structured exercise rehabilitation has gained substantial attention as a vital, non-pharmacological approach to enhancing both the physiological and psychological well-being of this clinical cohort. This systematic review aims to assess the efficacy of structured exercise regimens on functional output, physical capacity metrics, and health-related quality of life (HRQoL) among patients with cardiac implantable electronic devices (CIEDs), including conduction system pacing (CSP) (His bundle pacing (HBP), left bundle branch area pacing (LBBAP)), and cardiac resynchronization therapy (CRT).

A comprehensive literature retrieval was executed across electronic databases, including PubMed, Google Scholar, Scopus, ScienceDirect, and Web of Science, targeting research published from 2015 to 2026. This investigation considered randomized controlled trials (RCTs), clinical trials, comparative analyses, and observational research evaluating exercise-focused interventions in pacemaker and implantable device recipients. Methodological integrity and risk of bias were systematically evaluated using design-appropriate standardized critical appraisal frameworks. Eleven distinct studies fulfilled all specified selection criteria and were incorporated into the final analysis.

The synthesized evidence indicates that structured physical regimens comprising aerobic conditioning, resistance training, inspiratory muscle exercises, flexibility workouts, high-intensity interval training (HIIT), and remote or home-based telerehabilitation yielded substantial improvements in exercise endurance, cardiopulmonary fitness, breathing muscle function, and overall functional performance. Additionally, multiple investigations highlighted significant advancements in patient self-efficacy, psychological health, and HRQoL metrics. Home-centered and digitally monitored rehabilitation protocols demonstrated strong safety profiles, high feasibility, and excellent patient compliance rates. Methodologically, the majority of the reviewed literature exhibited moderate-to-high quality alongside a low-to-moderate risk of bias.

Tailored exercise rehabilitation protocols represent a safe and highly capable strategy for boosting functional performance, physical stamina, and life quality in individuals with cardiac physiological pacemakers and related implantable technologies. Holistic rehabilitation framework combinations incorporating aerobic and strength training, respiratory muscle development, psychosocial counseling, and customized pacing adjustments are essential to accelerating recovery, restoring confidence in physical activity, and maximizing long-term clinical success for this patient population.

## Introduction and background

Cardiac implantable electronic devices (CIEDs) are widely used in the management of bradyarrhythmias, conduction abnormalities, and heart failure. Recent advancements in pacing technology have led to the emergence of cardiac physiological pacing (CPP), which includes His bundle pacing (HBP), left bundle branch area pacing (LBBAP), and cardiac resynchronization therapy (CRT) [1]. Physiological pacing preserves normal ventricular activation pathways and reduces electrical dyssynchrony when compared with conventional right ventricular pacing [2]. As established by Cano et al. [3] and further contextualized by Sutton and Prakash (2025) [4], physiological pacing configurations utilize these natural conduction pathways to maintain ventricular synchrony, contrasting sharply with historical non-physiological pacing architectures of conventional right ventricular pacing.

Despite improvements in device implantation techniques and programming strategies, many patients continue to experience fatigue, reduced exercise tolerance, and limitations in activities of daily living following pacemaker implantation. Functional capacity is considered an important prognostic indicator in cardiovascular disease and is commonly assessed using outcome measures, such as peak oxygen uptake (VO₂ peak), the six-minute walk test (6MWT), metabolic equivalents (METs), and perceived exertion scales [5]. Conduction system pacing (CSP) has also been shown to improve cardiac function, myocardial work, and functional performance in patients with heart failure [6]. 

In addition to physical impairments, patients receiving CPP devices may experience anxiety, fear of movement, reduced confidence during physical activity, and social limitations, all of which can negatively affect health-related quality of life (HRQoL) [7]. Recent advances in CRT and CSP have demonstrated improvements in cardiac performance and symptom reduction; however, evidence regarding their influence on patient-reported quality of life outcomes remains limited [8]. 

Exercise rehabilitation is increasingly recognized as an important component of cardiovascular management. Structured rehabilitation programs incorporating aerobic training, resistance exercises, breathing exercises, flexibility training, and patient education can improve exercise tolerance, autonomic regulation, endothelial function, and peripheral muscle conditioning [9]. Studies have also suggested that combining exercise rehabilitation with pacing therapy may provide additional improvements in functional capacity and symptom management beyond device therapy alone [10]. 

Although the field of CPP is rapidly evolving, existing literature has mainly focused on implantation success, pacing parameters, echocardiographic remodeling, and heart failure outcomes. Limited evidence is available regarding the effectiveness of structured exercise rehabilitation on exercise capacity and HRQoL, specifically among patients with CPP devices. Furthermore, the combined effects of physiological pacing and rehabilitation-based interventions remain inadequately explored.

Therefore, the present study aims to evaluate the effect of a structured exercise rehabilitation program on exercise capacity and HRQoL among patients with CIEDs, including pacemakers, ICDs, and CRT devices. For the purpose of this review, the term physiological pacing encompasses both CSP and CRT, as both strategies aim to preserve or restore ventricular synchrony. Exercise capacity will be assessed using standardized functional outcome measures, while HRQoL will be evaluated using validated patient-reported questionnaires. The findings of this study may contribute toward the development of evidence-based rehabilitation strategies that complement physiological pacing and improve physical performance, functional independence, and overall quality of life in this patient population.

## Review

Methodology

Study Design

This systematic review was executed utilizing an evidence-based framework that aggregated data from clinical trials, randomized controlled trials (RCTs), observational research, cross-sectional investigations, and meta-analyses. Methodological rigor and risk of bias were systematically evaluated using the revised Cochrane risk of bias tool (RoB 2) for randomized designs, while the Appraisal Tool for Cross-Sectional Studies (AXIS) was applied to cross-sectional literature.

Given the substantial heterogeneity of the included literature regarding study designs (ranging from randomized trials to cross-sectional surveys), device architectures, and clinical endpoints, no quantitative statistical synthesis (e.g., meta-analysis or meta-regression) was performed. Instead, the gathered evidence is synthesized and presented purely descriptively via narrative analysis.

Search Strategy

A systematic and comprehensive literature search was conducted to identify studies investigating the effects of structured exercise interventions on functional capacity and HRQoL in patients with CIEDs, including pacemakers, implantable cardioverter-defibrillators (ICDs), and CRT devices. Electronic databases, namely PubMed, Scopus, Google Scholar, Web of Science, and ScienceDirect, were searched for articles published between 2015 and 2026. The final literature search was conducted on 30 April 2026. The complete, line-by-line search strategies and exact syntax utilized for all five electronic repositories are detailed comprehensively further.

Search terms were developed using a combination of Medical Subject Headings (MeSH) and free-text keywords related to cardiac implantable devices, exercise-based interventions, rehabilitation, functional performance, and quality of life outcomes. For PubMed, the following exact search string was applied to the title/abstract fields: ("Cardiac Physiological Pacing" OR "Cardiac Implantable Electronic Devices" OR Pacemaker OR ICD OR "Cardiac Resynchronization Therapy") AND (Exercise Rehabilitation OR Exercise OR "Physical Activity" OR "Aerobic Training" OR "Resistance Training" OR Rehabilitation) AND ("Quality of Life" OR "Exercise Capacity" OR "Functional Capacity" OR "Six Minute Walk Test" OR "Peak VO_^2^_"). For Scopus, this strategy was translated into field-specific terms targeting title, abstract, and keywords using the syntax: (TITLE-ABS-KEY("Cardiac Physiological Pacing" OR "Cardiac Implantable Electronic Devices" OR "pacemaker" OR "ICD" OR "Cardiac Resynchronization Therapy") AND TITLE-ABS-KEY("Exercise Rehabilitation" OR "Exercise" OR "Physical Activity" OR "Aerobic Training" OR "Resistance Training" OR "Rehabilitation") AND TITLE-ABS-KEY("Quality of Life" OR "Exercise Capacity" OR "Functional Capacity" OR "Six Minute Walk Test" OR "Peak VO_2_"))." To ensure completeness, database filters were restricted to "Human Subjects" and "English Language" without applying restrictive design filters during the initial screening phase, thereby maximizing retrieval sensitivity. In addition, reference lists of eligible studies were manually screened to identify further relevant publications. Only studies published in English were considered for inclusion.

To construct precise and comprehensive search strings, Boolean operators (AND, OR) were deployed to connect these terms. Additionally, the reference lists of all retrieved candidate articles were manually scrutinized to uncover further eligible studies. The scope of this review was restricted to investigations published exclusively in the English language.

Study Selection and Data Extraction Process

To minimize single-investigator bias and ensure methodological reproducibility, literature screening, study selection, and data extraction were performed independently by more than one reviewer. The author team collaboratively screened titles and abstracts against the predefined eligibility criteria, followed by an independent full-text review of potentially eligible studies. Baseline parameters - including sample sizes, specialized device configurations, FITT exercise metrics, and primary functional outcomes - were extracted using a standardized data abstraction form. Any emerging discrepancies or disagreements regarding study inclusion or data points were successfully resolved through collaborative team discussion and consensus-driven arbitration.

Literature screening and study selection were performed independently by two reviewers (K and P). Initial screening of titles and abstracts was followed by a strict dual full-text review. Any emerging discrepancies or disagreements regarding study inclusion or data points were successfully resolved through consultation and arbitration with a third independent reviewer (R) to achieve a consensus-driven resolution.

Inclusion Criteria

Studies were considered eligible for inclusion if they featured male or female cohorts aged between 52 and 74 years who had received a CIED, including pacemakers, ICDs, and CRT devices at least three months before the intervention. To meet the definition of a "structured exercise program," interventions were required to explicitly outline operational parameters across the frequency, intensity, time, and type (FITT) framework: type (aerobic conditioning, resistance training, HIIT, or inspiratory muscle training (IMT)); frequency (> two sessions per week); duration/time (> four weeks in program length); and defined Intensity metrics (e.g., % VO₂ peak, heart rate reserve, or Borg scale ratings). Crucially, due to fixed upper tracking rates or blunted chronotropic responses in pacemaker-dependent cohorts, traditional target heart rate (THR) prescriptions were classified as insufficient. Eligible studies were required to define exercise intensity using surrogate, chronotropically independent metrics, specifically the Borg Rating of Perceived Exertion (RPE scale 11-14), ventilatory thresholds, or percentages of peak oxygen consumption (% VO₂ peak). Studies assessing active rate-responsive functions (RRF) and metabolic sensor adjustments during rehabilitation were explicitly included to evaluate pacing-exercise interactions. Eligible candidates were required to be clinically stable under optimal pharmacological management at the time of study enrollment. Literature selection was restricted to peer-reviewed papers published up to April 2026 and indexed within online digital repositories such as PubMed, MEDLINE, PEDro, ResearchGate, and Google Scholar.

Exclusion Criteria

Exclusion criteria applied to studies involving patient cohorts with recent infectious diseases, a history of surgical operations within the three months preceding pacemaker placement, or individuals demonstrating a baseline functional capacity exceeding 85% of their predicted maximum walking distance. Additionally, trials involving patients with a terminal diagnosis (predicted survival under 12 months) or severe psychiatric disorders that could jeopardize study compliance, participation safety, or clinical follow-up were excluded. Finally, any literature failing to fulfill the explicit, predefined selection criteria regarding diagnostic methodologies and therapeutic approaches was omitted from the final analysis.

Quality Assessment

The methodological integrity of the included literature was evaluated using standardized risk-of-bias frameworks tailored to individual study architectures. To ensure objectivity and eliminate evaluation bias, the quality assessment was performed independently by two reviewers. Any emerging discrepancies or scoring disagreements regarding specific domains were successfully resolved through a structured consensus procedure involving collaborative team discussion.

For RCTs appraised via the RoB 2, the overall risk-of-bias judgment for each study was determined strictly according to the official Cochrane signaling-question algorithm. A study was judged to have "some concerns" if it presented some concerns in at least one domain but no high-risk signals, while a "low risk" overall judgment required low-risk evaluations across all five domains. For cross-sectional studies evaluated through the AXIS, overall quality profiles were synthesized based on the cumulative reporting clarity and internal consistency of the 20 checklist criteria.

While achieving complete assessor and participant blinding presented a logistical hurdle across several protocols, the analyzed RCTs generally presented negligible risk regarding their randomization sequences and outcome reporting. For the cross-sectional studies, minimal bias risks were identified, despite minor limitations related to population representativeness and non-response factors. Cumulatively, the selected literature displayed moderate-to-high methodological quality, confirming its suitability for synthesis in this review.

*Publication Bias* Although this systematic review includes 11 studies, a quantitative meta-analysis was not performed due to the substantial heterogeneity in study designs, device configurations, and outcome metrics. Consequently, a Funnel Plot and Egger’s linear regression test were omitted, as these statistical assessments of publication bias are strictly reserved for quantitative meta-analyses and lack an appropriate framework and power in qualitative systematic syntheses.

**Figure 1:**
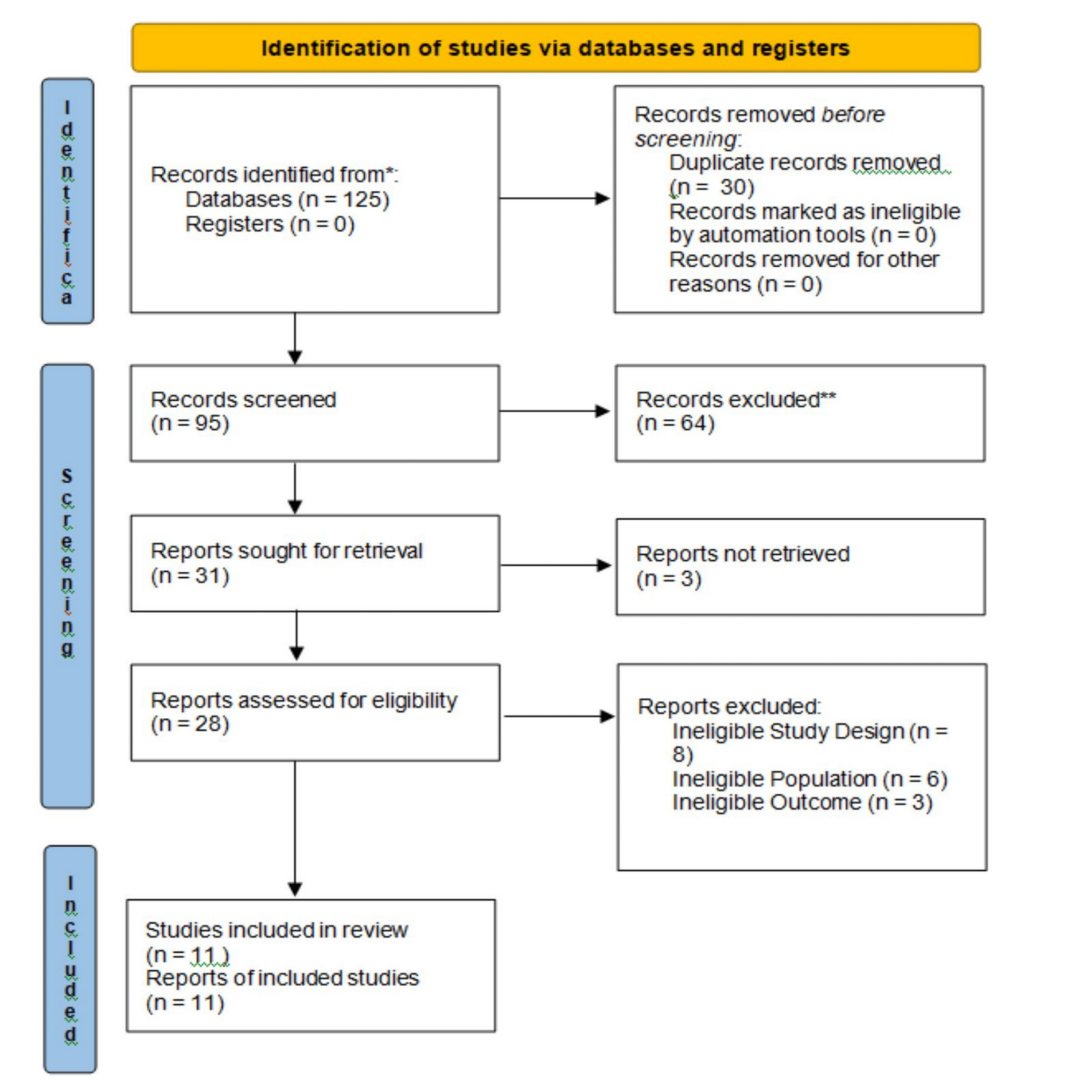
PRISMA 2020 flow diagram of study selection process PRISMA: Preferred Reporting Items for Systematic Reviews and Meta-Analyses

A total of 125 records were identified through database searching, n = 125, and registers, n = 0. After removing 30 duplicate records, 95 records remained for screening. Of these, 64 records were excluded based on screening. Thirty-one reports were sought for retrieval, of which three reports could not be retrieved. The remaining 28 full-text articles were assessed for eligibility. After excluding 17 reports due to ineligible study design (n = 8), ineligible population (n = 6), and ineligible outcome measures (n = 3), 11 studies met the inclusion criteria and were included in the systematic review, as given in Figure 1.

Results

A comprehensive synthesis of the literature resulted in the inclusion of 11 unique investigations. These selected studies offer a detailed, multi-dimensional overview of contemporary therapeutic approaches, rehabilitation frameworks, and functional assessment methodologies applicable to individuals managed with CPP and related implantable electronic hardware.

This study summarizes recent studies evaluating rehabilitation strategies, exercise interventions, and quality of life outcomes in patients with CIEDs such as pacemakers, ICDs, and CRT devices. The findings indicate that interventions including HIIT, IMT, home-based exercise, and telemonitored rehabilitation are generally safe and effective in improving exercise capacity, respiratory function, physical activity, and overall quality of life. Observational studies also highlight the influence of psychosocial factors, frailty, and self-efficacy on patient outcomes, as given in Table 1.

**Table 1:** Rehabilitation and quality of life studies in patients with cardiac implantable devices VO₂: peak oxygen consumption; HIIT: high-intensity interval training; IMT: inspiratory muscle training; LVEF: left ventricular ejection fraction; NT-proBNP: N-terminal pro-b-type natriuretic peptide; CRT: cardiac resynchronization therapy; SF-36: 36-item short form survey; EQ-5D-5L: EuroQol 5-Dimension 5-Level scale; NYHA: New York Heart Association; ICD: implantable cardioverter-defibrillator

Author(s)	Year	Study design	Sample size	Outcome measures	Interventions	Results	Conclusion
Nyman et al. [10]	2026	Randomized controlled trial	56 (HIIT n = 28, control n = 28)	Peak VO₂, quality of life, ventricular arrhythmias	High-intensity interval training (HIIT)	Improved peak VO₂ and quality of life without increasing ventricular arrhythmias	HIIT appears safe and effective in selected patients with ICD
Bellur et al. [11]	2026	Randomized controlled trial	32 (19 IMT, 13 control)	Maximal inspiratory pressure, LVEF, NT-proBNP, 6-minute walk distance, daily activity limitation, quality of life	12-week home-based inspiratory muscle training (IMT) vs. usual care	Significant improvements in respiratory muscle strength, cardiac function, exercise capacity, daily activities, and quality of life; NT-proBNP decreased	Home-based IMT is a simple and effective adjunct rehabilitation strategy for CRT patients with heart failure
Safdar et al. [12]	2026	Randomized trial	23 (multi-point pacing n = 14, Single-point pacing n = 9)	Exercise capacity, force-frequency relationship	Multi-point pacing vs single-point pacing	Multi-point pacing was associated with better exercise performance and improved contractile response	Multi-point pacing may enhance functional capacity compared with single-point pacing in CRT recipients
Stavrou et al. [13]	2026	Cross-sectional study	120	Quality of life assessed by SF-36 and EQ-5D-5L	None (observational)	Quality of life was significantly associated with NYHA class, comorbidities, pacing mode, and patient information level	Multiple clinical and psychosocial factors influence quality of life in pacemaker recipients
Tsoni et al. [14]	2026	Cross-sectional study	112 (permanent pacemaker recipients n = 112)	Quality of life before and at 6 and 12 months after implantation	None (observational)	Quality of life improved progressively after pacemaker implantation	Permanent pacemaker implantation is associated with sustained quality-of-life improvements
Yu et al. [15]	2026	Cross-sectional study with latent profile and mediation analysis	132	Kinesiophobia, exercise self-efficacy, and physical activity	None (observational)	Exercise self-efficacy mediated the negative effect of kinesiophobia on physical activity	Interventions targeting fear of movement and self-efficacy may improve physical activity levels
Katayıfçı et al. [16]	2022	Randomized controlled trial	36	6-minute walk test, respiratory muscle strength and endurance, quality of life	Inspiratory muscle strength training vs inspiratory muscle endurance training	Both interventions improved exercise capacity, respiratory muscle performance, and quality of life	Inspiratory muscle training is beneficial in pacemaker patients with heart failure
Kramer et al. [17]	2017	Cross-sectional study	219	Frailty, physical activity, mobility	None (observational)	Frail patients demonstrated lower physical activity and reduced mobility	Frailty is an important determinant of functional status in patients with cardiac implantable electronic devices
Lau et al. [18]	2016	Randomized controlled trial	43	Physical function, symptoms, adherence	Early home-based walking program	Improved functional capacity and symptoms with no adverse events	Early home-based walking is safe and beneficial after initial ICD implantation
Piotrowicz et al. [19]	2015	Randomized controlled trial	111	Peak VO₂, 6-minute walk test, quality of life, safety	Home-based telemonitored Nordic walking	Significant improvements in exercise capacity and quality of life with no major adverse events	Telemonitored home exercise is safe, feasible, and effective in heart failure patients with cardiac devices
Berg et al. [20]	2015	Randomized clinical trial	196 (comprehensive cardiac rehabilitation group n = 98, Control group n = 98)	Physical and psychological outcomes	Thorough cardiac rehabilitation vs usual care	Significant improvements in rehabilitation outcomes	Thorough cardiac rehabilitation improves outcomes in ICD patients

Assessment of Risk of Bias

Each selected study underwent a comprehensive risk-of-bias assessment to ensure the validity and reliability of the synthesized findings. RCTs were assessed using the RoB 2 tool [21], whereas observational and cross-sectional studies were evaluated using the AXIS [22]. Based on the RoB 2 criteria, the majority of the selected literature exhibited a moderate risk profile, primarily driven by reporting practices, randomization protocols, and missing outcome data. Furthermore, lack of blinding presented persistent challenges across these trials, particularly regarding deviations from intended interventions and outcome measurements. Consequently, the overall risk of bias across the RCTs fell under the "some concerns" category, reflecting acceptable baseline methodological quality tempered by vulnerability to detection and performance biases.

Table 2 presents the risk of bias assessment of the included randomized studies across key methodological domains, including randomization, intervention deviations, missing data, outcome measurement, and reporting bias. Most studies demonstrated low risk in randomization and missing outcome data, while several showed some concerns regarding intervention adherence and outcome measurement, resulting in an overall judgment of “some concerns” for the majority of studies.

**Table 2:** Risk of bias assessment using the RoB2 tool RoB 2: revised Cochrane risk of bias tool; RCT: randomized controlled trial

Study	Bias arising from the randomization process	Bias due to deviations from intended interventions	Bias due to missing outcome data	Bias in the measurement of the outcome	Bias in the selection of the reported result	Overall risk of bias
Nyman et al. [10]	Low risk	Some concerns	Low risk	Some concerns	Low risk	Some concerns
Bellur et al. [11]	Low risk	Some concerns	Low risk	Some concerns	Low risk	Some concerns
Safdar et al. [12]	Low risk	Low risk	Low risk	Low risk	Some concerns	Low risk to some concerns
Katayıfçı et al. [16]	Low risk	Some concerns	Low risk	Some concerns	Low risk	Some concerns
Lau et al. [18]	Low risk	Some concerns	Low risk	Some concerns	Low risk	Some concerns
Piotrowicz et al. [19]	Low risk	Some concerns	Low risk	Low risk	Low risk	Some concerns
Berg et al. [20]	Low risk	Some concerns	Low risk	Some concerns	Low risk	Some concerns

Table 3 presents the AXIS quality appraisal of the included cross-sectional studies. Overall, the studies demonstrated clear objectives, appropriate study designs, valid outcome measurements, and adequate statistical reporting. However, common methodological limitations included a lack of representative sampling, absence of strategies to address non-response bias, and limited information regarding non-respondents, which may affect the generalizability of the findings.

**Table 3:** Quality assessment of included cross-sectional studies using the AXIS tool AXIS: Appraisal Tool for Cross-Sectional Studies

AXIS appraisal question	Stavrou et al. (2026) [13]	Tsoni et al. (2026) [14]	Yu et al. (2026) [15]	Kramer et al. (2017) [17]
Were the aims/objectives of the study clear?	Yes	Yes	Yes	Yes
Was the study design appropriate for the aims?	Yes	Yes	Yes	Yes
Was the sample size justified?	Yes	Yes	Yes	No
Was the target population clearly defined?	Yes	Yes	Yes	Yes
Was the sampling frame appropriate?	No	No	No	No
Was the selection process representative?	No	No	No	No
Were measures taken to address non-responders?	No	No	No	No
Were the outcome variables measured appropriately?	Yes	Yes	Yes	Yes
Were the tools piloted/published previously?	Yes	Yes	Yes	Yes
Was statistical significance clearly determined?	Yes	Yes	Yes	Yes
Were the methods described in enough detail?	Yes	Yes	Yes	Yes
Were the basic data adequately described?	Yes	Yes	Yes	Yes
Is there concern regarding non-response bias?	Yes	Yes	Yes	Yes
Was the info about non-respondents described?	No	No	No	No
Were the results internally consistent?	Yes	Yes	Yes	Yes
Were all method-defined results presented?	Yes	Yes	Yes	Yes
Were the discussions and conclusions justified?	Yes	Yes	Yes	Yes
Were the study limitations discussed?	Yes	Yes	Yes	Yes
Were funding/conflicts of interest reported?	Yes	Yes	Yes	Yes
Was ethical approval/informed consent attained?	Yes	Yes	Yes	Yes

Discussion

This systematic investigation assessed how structured physical training regimens impact functional performance indicators and HRQoL metrics in individuals managed with CIEDs. Synthesized data across the compiled literature indicate that structured physical conditioning drives substantial improvements in aerobic endurance, muscular power, flexibility, physical motility, and global quality-of-life scores. These outcomes strongly reinforce an expanding body of clinical evidence confirming that structured physical rehabilitation is both highly tolerable and highly therapeutic for patients utilizing advanced CSP mechanisms.

Following CIED implantation, patients frequently struggle with exercise limitations, deconditioning, and kinesiophobia. However, the synthesized evidence demonstrates that the clinical impact of structured exercise rehabilitation is heavily dependent on the specific device profile:

Pacemakers (CSP/CPP): Interventions primarily optimize daily operational tolerance, chronotropic responses, and respiratory mechanics, countering baseline myocardial dysfunction.

ICDs: Programs fundamentally target the mitigation of kinesiophobia, psychosocial maladaptation, and fear of device discharges, proving that high-intensity tracking can be safely introduced without triggering ventricular arrhythmias.

CRT: Rehabilitation acts synergistically with multi-point pacing configurations to aggressively drive left ventricular remodeling, maximize ventilatory efficiency, and improve functional outcomes in severe heart failure cohorts.

A key physiological finding across the synthesized literature is that traditional THR prescriptions are fundamentally flawed for pacemaker-dependent individuals. Because these patients exhibit blunted chronotropic responses or are bound by fixed upper tracking rates programmed into their devices, relying on standardized THR formulas risks over- or under-prescribing exercise workloads. The reviewed evidence demonstrates that successful cardiac rehabilitation programs bypass this limitation by utilizing subjective exertion profiling (Borg RPE) and metabolic metrics (peak VO₂) to safely guide training intensity without causing tracking conflicts or device-mediated asynchronous pacing.

Collective Synthesis of Cardiopulmonary and Ventilatory Adaptations

Across the compiled literature, structured physical training universally improved aerobic endurance and functional mobility, though through different physiological pathways depending on the modality. Aerobic and HIIT tracks optimized cardiovascular stamina and peak oxygen consumption VO₂ safely across both pacemakers and aggressive ICD cohorts, demonstrating no adverse rhythm events. Concurrently, targeted respiratory interventions, specifically home-directed IMT, served as a highly effective mechanism to directly reduce dyspnea and maximize ventilatory mechanics. This respiratory benefit was particularly pronounced in patients with heart failure utilizing CRT devices, where respiratory conditioning paired with resynchronization therapy directly advanced peripheral muscle conditioning and overall life-satisfaction scores.

Several investigations identified a clear nexus connecting physical activity levels, frailty indices, and perceived quality of life in device recipients. Kramer et al. demonstrated that physical frailty and restricted mobility directly correlated with diminished daily activity and lower functional scores in individuals with electronic cardiac implants [17]. Concurrently, Yu et al. discovered that kinesiophobia and low exercise self-efficacy heavily restricted physical activity engagement among permanent pacemaker users [15]. These insights confirm that tailored rehabilitation tracks are crucial not just for restoring bodily movement but also for breaking down psychological reservations and reinforcing patient confidence during physical tasks.

Consistently elevated scores in HRQoL across the selected literature underscore a multi-dimensional recovery pattern that spans physical, emotional, and social domains. Rather than experiencing stagnant outcomes, patients demonstrate a progressive, long-term upward trajectory in global life satisfaction following device implantation, with the most pronounced improvements tied to the adoption of healthy behaviors and active participation in structured physical conditioning [14]. From a psychosocial standpoint, comprehensive cardiac rehabilitation serves as a powerful mechanism to bolster emotional stability, restore community reintegration, and build psychological resilience, effectively tackling baseline fears and kinesiophobia [13,20]. Ultimately, these findings emphasize that therapeutic exercise acts as more than a tool for physical restoration; it functions as a holistic intervention that directly improves emotional balance and social adaptation.

Device Programming and Rate-Responsive Functions (RRF)

From a device programming standpoint, Safdar et al. offered relevant insight into how pacing strategies interface with exercise outcomes by contrasting multi-point and single-point pacing tracks [12]. Their data revealed clear differences in exercise endurance and force-frequency dynamics between the two setups, proving that combining customized pacing optimization with targeted physical training can further elevate physical capacity. This highlights the value of pairing individualized device configuration with customized physical protocols to optimize cardiac work efficiency.

Work by Caloian et al. [23] and Świerżyńska et al. [24] highlights that rate-responsive functions (RRF) must be tailored to individual metabolic demands during physical exertion. These systems rely on embedded pacing sensors, such as accelerometers (detecting physical motion) and minute ventilation sensors (detecting respiratory rate changes), to dynamically scale the pacing rate. Without precise sensor calibration before rehabilitation, patients may experience chronotropic incompetence or inappropriate pacing, limiting the therapeutic efficacy of aerobic conditioning.

Despite these encouraging perspectives, various limitations must be highlighted. There was substantial heterogeneity across the selected studies concerning exercise design, workload intensity, intervention timelines, baseline patient health profiles, and chosen endpoint metrics. Additionally, the inclusion of cohorts with distinct electronic implants ranging from standard single/dual-chamber pacemakers to complex implantable defibrillators and resynchronization therapy configurations makes direct cross-study comparisons challenging. Many of the reviewed trials relied on relatively constrained sample sizes, which may restrict the broader applicability of their conclusions. Achieving strict double-blinding is naturally difficult in exercise-based trials, leaving open a potential risk for performance bias. Lastly, the relative scarcity of extended, longitudinal data leaves long-term functional maintenance partially unverified.

Nevertheless, the baseline methodological execution across the reviewed literature proved moderate to high, with the vast majority of studies tracking favorable returns from structured exercise programs [21,22]. The overall evidence demonstrates that multi-faceted rehabilitation frameworks comprising aerobic workloads, breathing exercises, resistance training, and home-based tracks are structurally safe, practical, and highly efficient for optimizing physical function and emotional well-being in patients with physiological pacemakers.

Future research should focus on implementing large-scale, multicenter randomized trials that utilize standardized training protocols and expanded monitoring timelines to formulate definitive, evidence-based exercise guidelines for pacing recipients. Furthermore, trials exploring the combined impact of physical training, psychosocial counseling, precise pacing customization, and digital tele-rehabilitation will be instrumental in building complete care programs designed to fully restore functional capacity and maximize quality of life in this population.

## Conclusions

Structured exercise rehabilitation programs appear to be safe and potentially beneficial for improving functional capacity, exercise tolerance, and HRQoL in individuals with CIEDs. Multicomponent interventions incorporating aerobic exercise, resistance training, IMT, flexibility exercises, and home-based rehabilitation were associated with favorable effects on cardiopulmonary fitness, physical function, and psychological well-being across the included studies. However, considerable heterogeneity in study designs, exercise protocols, device types, and outcome measures limits the strength of the conclusions. Therefore, the current evidence should be considered supportive rather than definitive. Further large-scale, multicenter randomized studies employing standardized rehabilitation protocols and longer follow-up periods are required to establish evidence-based recommendations for this population.
